# Dyslipidemia in diffuse large B-cell lymphoma based on the genetic subtypes: a single-center study of 259 Chinese patients

**DOI:** 10.3389/fonc.2023.1172623

**Published:** 2023-06-09

**Authors:** Yi Xu, Huafei Shen, Yuanfei Shi, Yanchun Zhao, Xiaolong Zhen, Jianai Sun, Xueying Li, De Zhou, Chunmei Yang, Jinhan Wang, Xianbo Huang, Juying Wei, Jian Huang, Haitao Meng, Wenjuan Yu, Hongyan Tong, Jie Jin, Wanzhuo Xie

**Affiliations:** Department of Hematology, The First Affiliated Hospital, College of Medicine, Zhejiang University, Hangzhou, Zhejiang, China

**Keywords:** diffuse large B-cell lymphoma, molecular typing, dyslipidemia, hypertriglyceridemia, BN2 subtype, MCD subtype, EZB subtype, A53 subtype

## Abstract

**Background:**

Diffuse large B-cell lymphoma (DLBCL) is a kind of highly heterogeneous non-Hodgkin lymphoma, both in clinical and genetic terms. DLBCL is admittedly categorized into six subtypes by genetics, which contain MCD, BN2, EZB, N1, ST2, and A53. Dyslipidemia is relevant to a multitude of solid tumors and has recently been reported to be associated with hematologic malignancies. We aim to present a retrospective study investigating dyslipidemia in DLBCL based on the molecular subtypes.

**Results:**

This study concluded that 259 patients with newly diagnosed DLBCL and their biopsy specimens were available for molecular typing. Results show that the incidence of dyslipidemia (87.0%, p <0.001) is higher in the EZB subtype than in others, especially hypertriglyceridemia (78.3%, p = 0.001) in the EZB subtype. Based on the pathological gene-sequencing, patients with BCL2 gene fusion mutation are significantly correlative with hyperlipidemia (76.5%, p = 0.006) and hypertriglyceridemia (88.2%, p = 0.002). Nevertheless, the occurrence of dyslipidemia has no remarkable influence on prognosis.

**Conclusion:**

In summary, dyslipidemia correlates with genetic heterogeneity in DLBCL without having a significant influence on survival. This research first connects lipids and genetic subtypes in DLBCL.

## Introduction

Diffuse large B-cell lymphoma (DLBCL) is characterized by aggressiveness and heterogeneity epigenetically and genetically. Besides, DLBCL accounts for approximately 35% of non-Hodgkin lymphoma (NHL) and is the most common lymphoma in adults (1). In 2018, the definition of molecular typing was initially propounded. Shipp et al. classified DLBCL into clusters 1–5 by genetic abnormality (2). Staudt et al. implemented molecular typing by analyzing genetic alterations and proposed four prominent subtypes: MCD (featured as the co-occurrence of *MYD88 L265p* and *CD79B* mutations), BN2 (featured as *BCL6* fusion mutations and *NOTCH*2 mutations), EZB (featured as *EZH2* mutations and *BCL2* fusion mutations), and N1 (featured as *NOTCH1* mutations). Later, two other types were added, including ST2 (featured as *SGK1* and *TET2* mutations) and A53 (featured as *TP53* aneuploid mutations) (3, 4). Researchers had considered the influence of *MYC* mutations and therefore divided the EZB subtype into *MYC*-positive EZB isoforms and *MYC*-negative EZB isoforms (4). While the prior simple algorithm of six subtypes is more frequently being applied in clinical. Dyslipidemia has been considered correlative to multitudes of solid tumors, like breast cancer, prostate cancer, thyroid cancer, colorectal cancer, and lung cancer (5–9). Recently, researchers have found that a portion of hematologic malignancies is relative to the incidence of dyslipidemia, for instance, chronic leukemia and acute promyelocytic leukemia (10, 11). Ma et al. recently found that cholesterol biosynthesis relative axis may have an oncogenic activation in DLBCL with *BCL2*-*IGH* translocations (12). While the correlation between dyslipidemia and DLBCL has not been reported, especially on the genetic aspect. Therefore, we aim to investigate dyslipidemia in DLBCL in the context of molecular typing and explore the correlation between dyslipidemia and gene mutations.

In this retrospective study, we analyzed 259 DLBCL patients with gene sequencing to assess dyslipidemia in newly diagnosed DLBCL.

## Methods

### Patients and sample collection

A total of 259 newly diagnosed DLBCL patients were selected with indispensable pathological issues to analyze molecular typing. All patients were admitted to the clinic center affiliated with the First Affiliated Hospital of Zhejiang University School of Medicine from April 2014 to November 2022. A total of 121 genes covering exons, fusion-relevant introns and alternative splicing areas are analyzed. Six typing methods were applied in this study, which contain MCD, BN2, EZB, N1, ST2, and A53. In terms of dyslipidemia, we collected lipid indices, including triglycerides (TG), total cholesterol (TC), high-density lipoprotein cholesterol (HDL-C), and low-density lipoprotein cholesterol (LDL-C). Lipid profiles were stratified according to *The Guidelines on Prevention and Treatment of Blood Lipid Abnormality in Chinese Adults (Version 2016)* (13). Dyslipidemia is defined as a disease with an abnormality in lipids, usually with increased levels of TC and TG. Dyslipidemia is classified into different subtypes in clinical aspects, pathogenesis, and physical-chemistry aspects. According to the WHO criteria developed by Fredrickson, which discriminate by physical and chemical features. Dyslipidemia is categorized into six types (14). Due to the complexity of this classification, another standard is rather easier. Dyslipidemia is defined into four types: hypertriglyceridemia, hypercholesterolemia, hypoalbuminemia, and mixed hyperlipidemia (both hypertriglyceridemia and hypercholesterolemia). This method is now used more frequently in clinics, and in our study, this method was applied (15, 16).

All patients were initially treated with the first-line therapy recommended by the National Comprehensive Cancer Network (NCCN) (17). Patients with CD20 positivity were primely treated with rituximab in combination with cyclophosphamide, doxorubicin, vincristine, and prednisone (R-CHOP) or R-CHOP-like regimens. Older patients were medicated with a dose-decreased R-CHOP regimen. The R-DA-EPOCH regimen was administered to patients with poor left ventricular functions.

Diagnosis and prognosis indicators related to DLBCL were collected and concluded as follows: B symptoms, Ann Arbor stage, international prognosis index (IPI), Eastern Cooperative Oncology Group stage (ECOG stage), and results of immunohistochemistry (IHC). We also collected demographic and other clinical data from medical records that concluded: age, sex, height, weight, body mass index (BMI), white blood count (WBC), hemoglobin count, platelet counts, albumin, alanine transaminase (ALT), serum creatinine, lactate dehydrogenase (LDH), ferritin, C-reactive protein (CRP), β microglobulin (β-MG), fibrinogen, prothrombin time (PT), activated prothrombin time (APTT), thrombin time (TT), International normalized ratio (INR), D-dimer, and cytokines including interleukin-2 (IL-2), interleukin-4 (IL-4), interleukin-6 (IL-6), interleukin-10 (IL-10), interleukin-17α (IL-17α), tumor necrosis factor-α (TNF-α), and interferon-γ (IFN-γ). We collected a total of 22,375 samples.

The study was approved by the institutional Ethics Committee and conducted in accordance with the Declaration of Helsinki.

### Classification of abnormality

According to the Guidelines on Prevention and Treatment of Blood Lipid Abnormality in Chinese Adults (Version 2016), we define dyslipidemia as hypertriglyceridemia, hypercholesterolemia, hypoalbuminemia, and mixed hyperlipidemia, which meets both hypertriglyceridemia and hypercholesterolemia.

The upper limits of normal TG, TC, and LDL were 1.70 mmol/L (150 mg/dl), 5.86 mmol/L (226.6 mg/dl), and 3.29 mmol/L (127.2 mg/dl), respectively. The lower limit of normal HDL was 0.78 mmol/L (30.2 mg/dl).

### Definition of molecular subtypes

**Figure 1:**
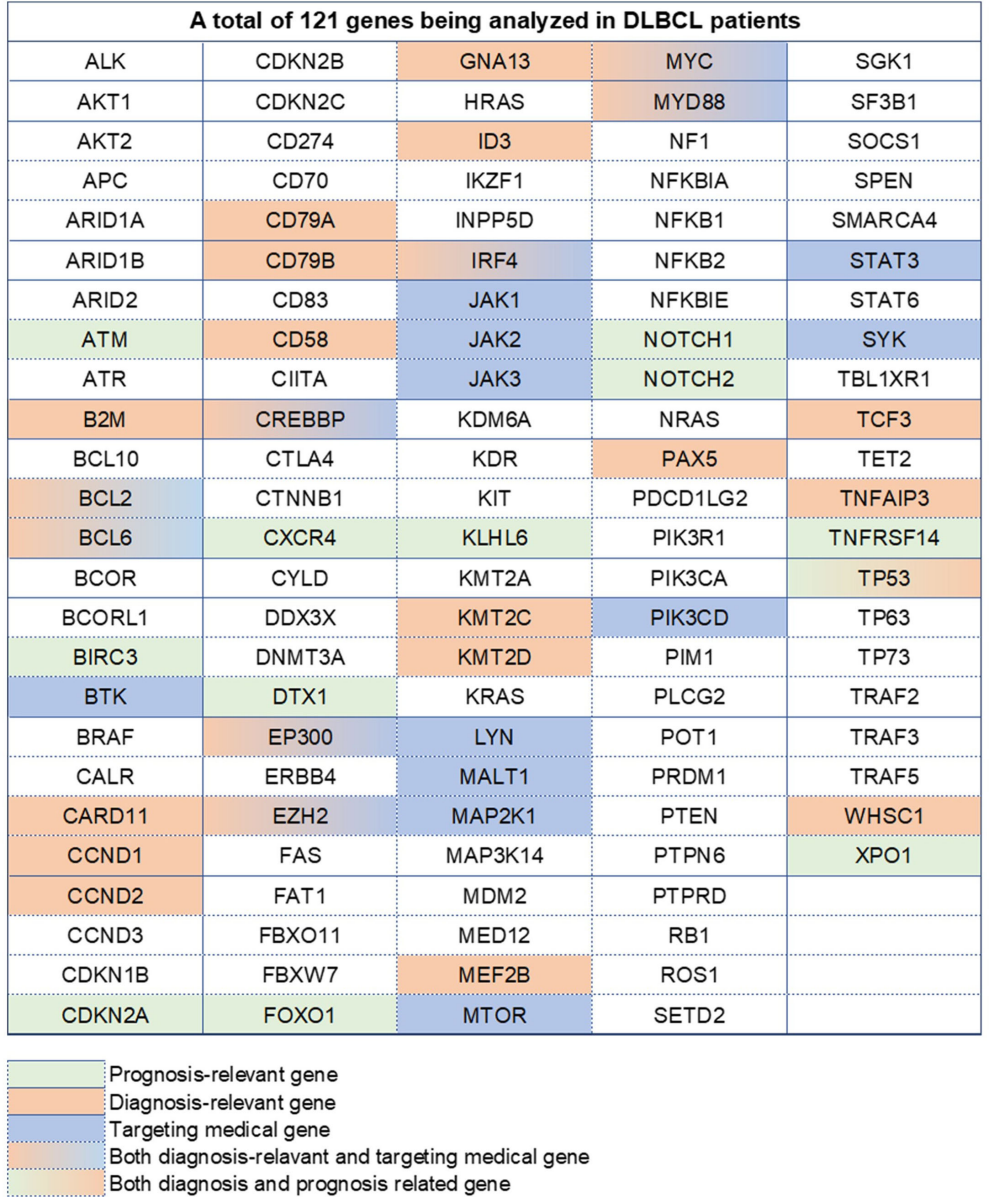
A total of 121 genes analyzed in DLBCL patients. Different colors represent different types, containing prognosis-relevant, diagnosis-relevant, and targeting medical-relevant genes.

Biopsy samples and tissue sections from pathological entities, such as swollen lymph nodes and gastrointestinal tissues, were saved for analysis at the institute. The testing platform was the Hiseq 4000 NGS platform (Illumina system), and the testing method was high-throughput sequencing, also called next-generation sequencing (NGS), contrary to the reference genome of GRCh37/hg19. This analysis covered 121 genes including exonic areas, intronic areas related to fusion mutations, and alternative splicing sites (3, 4). All these 121 genes are recorded in **Figure 1** and **Table S1**. There were 2,876 cases of gene abnormalities occurring in 259 patients, including fusion mutations, copy number variation, gene amplification, missense variation, nonsense variation, frameshift insertion/deletion, and non-frameshift insertion/deletion. Besides, 2,790 protein-alternative mutations were found based on the analysis. The LymphGen algorithm was applied in the analysis for DLBCL subtype prediction with probability. The probability was analyzed in the Naïve Bayes algorithm [4]. All information was collected from the clinical center. All these gene abnormalities are listed in **Table S2**.

### Statistical analysis

Data are presented as medians, absolute ranges, or frequencies. The Kruskal–Wallis H test was used to compare the distribution of numerical variables. The χ^2^ test was used for qualitative variables. The relationships between clinical factors and dyslipidemia were assessed using univariable and multivariable logistic regression models. All multivariable analyses were adjusted for sex and age. The Kaplan–Meier method was used to estimate univariate survival curves, and the differences between curves were calculated via the log-rank test. Multivariable Cox proportional hazard regression models were used to assess the prognostic impact of hypertriglyceridemia with regard to overall survival (OS) and progress-free survival (PFS). Statistical analyses were performed using SPSS software (version 23.0) and GraphPad Prism (version 9.5). P-values below 0.05 were considered statistically significant.

## Results

### Patient characteristics

**Figure 2:**
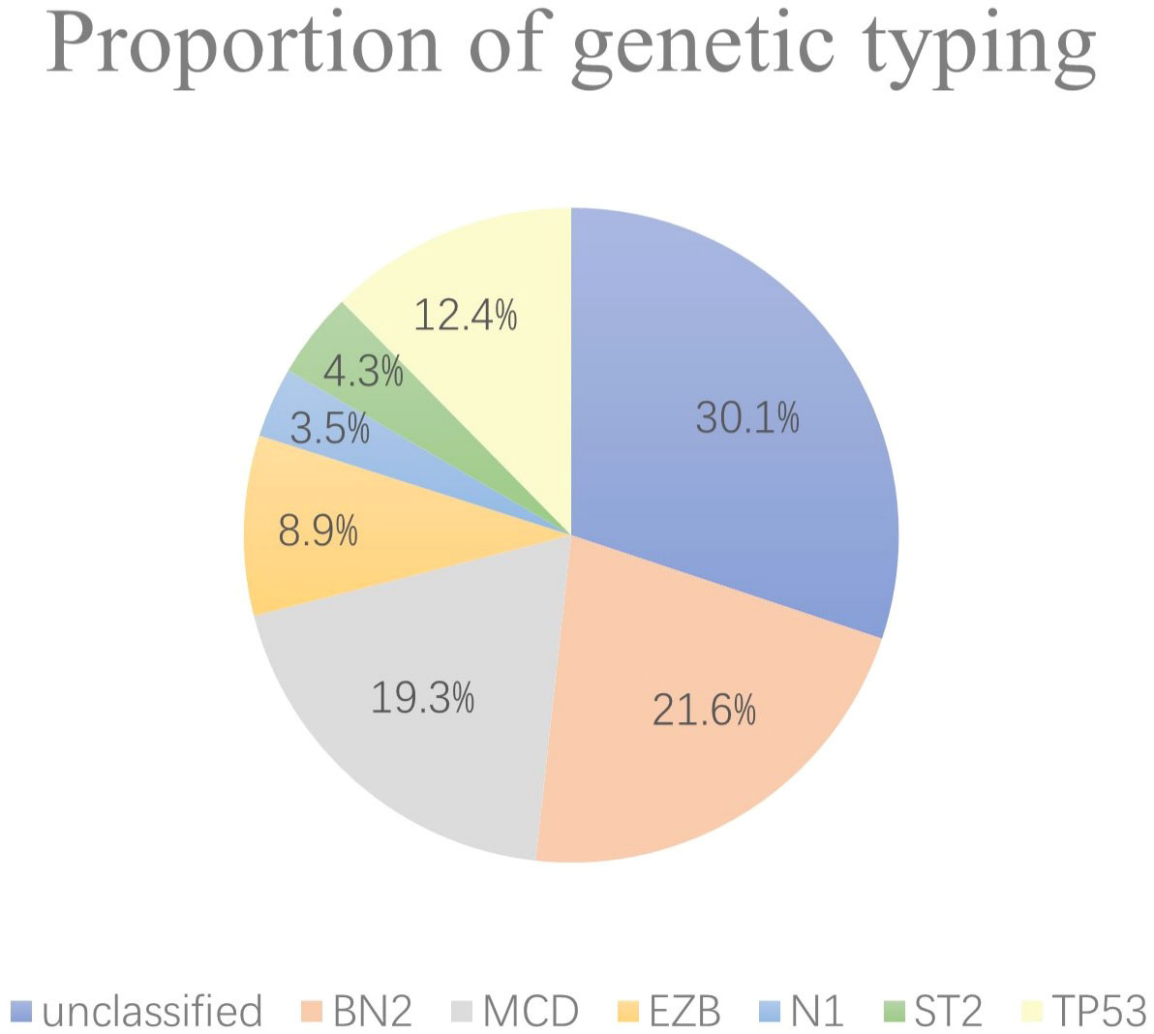
Proportion of genetic typing. In the six subgroups divided by genetics, the BN2 subtype, MCD subtype, and EZB subtype account for most, while the N1 and ST2 subtypes account for no more than 10% of the total.

**Figure 3:**
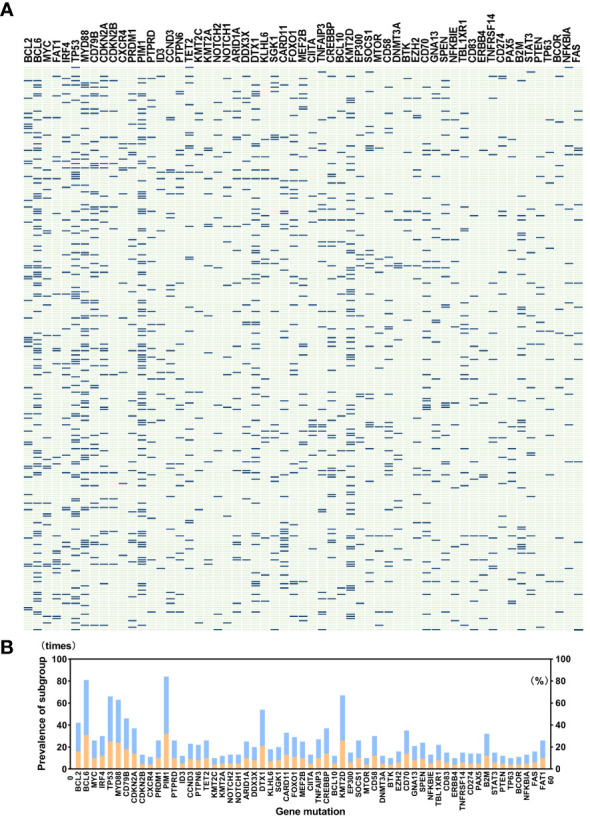
**(A)** shows gene mutations in detail in a total of 259 patients, and **(B)** indicates the prevalence of gene mutations. A total of 121 genes covering exon, fusion-relevant intron, and alternative splicing are analyzed, and we selected 60 types of gene mutations that occurred rather frequently or were molecular typing-related, like *BCL2*, *BCL6*, *MYC*, *MYD88*, *CD79B*, *PIM1*, *KMT2D*, *DTX1*, *TET2*, and *SGK1*. Panel **(B)** illustrates the prevalence of gene mutations; the blue band in the histogram represents mutation times, and the orange band shows percentage. Abnormalities of *BCL6*, *TP53*, *PIM1*, *MYD88*, and *KMT2D* occurred in more than 20% of patients, and *PIM1* mutations appeared most common.

A total of 259 newly diagnosed DLBCL patients are categorized into six genetic subtypes, and the proportion of each type is 21.6% MCD subtype, 19.3% BN2 subtype, 8.9% EZB subtype, 3.5% N1 subtype, 4.2% ST2 subtype, and 12.4% A53 subtype, respectively (**Figure 2**). Besides, 30.1% of patients are unclassified, and this isoform is defined as a black control. To investigate the concrete genes influencing the lipids, we considered 60 familiar decisive molecular typing genes such as *BCL2*, *BCL6*, *TP53*, *MYD88*, *MYC*, *NOTCH2*, and *EZH2*. More details regarding genetic typing and gene translocation are shown in **Figure 3**.

We recorded and analyzed the initial concentration of lipids before DLBCL patients received any treatment. Results show that TG in the EZB subtype was higher than in the unclassified subtype (Kruskal–Wallis H test, p = 0.001). The median concentration of TG in these two groups is 2.01 mmol/L (range 1.76–2.48 mmol/L) and 1.16 mmol/L (range 0.84–1.58 mmol/L), respectively. More importantly, results show that in the EZB subtype, the incidence of hypertriglyceridemia is 78.3% compared with 19.2% in the unclassified subtype, 36.0% in the MCD subtype, and 18.2% in the ST2 subtype (chi-square test and Fisher’s exact test, p <0.001, = 0.001, <0.001). In addition, dyslipidemia is discovered more commonly in the EZB subtype than in the unclassified subtype, with a significant statistical difference (87% vs 39.7%, p <0.001). The other three types of lipids (TC, HDL-C, and LDL-C), meanwhile, demonstrated no significant differences among the seven types.

According to the World Health Organization (WHO) classification of lymphoma, researchers put forward the concept of high-grade B-cell lymphoma based on three types of gene translocation: *BCL2*, *BCL6*, and *MYC*. Thus, we also analyzed the correlation between dyslipidemia and gene translocation. Based on pathological gene-sequencing, patients with *BCL2* gene fusion mutation are significantly more likely to have dyslipidemia (chi-square test, 76.5%, p = 0.006) and hypertriglyceridemia (chi-square test, 88.2%, p = 0.002) than patients without rearrangement of these three genes.

Other demography and clinical characteristics are demonstrated in **Table 1**.

**Table 1:** Characteristics of democracy and clinical factors in different types of DLBCL.

	Genetic typing							
	MCD n=50	BN2 n=56	EZB n=23	N1 n=9	ST2 n=11	A53 n=32	Unclassified n=78	P value
Age (years), range	66 (47-90)	62 (23-87)	60 (39-81)	70 (60-79)	69 (31-87)	64 (18-90)	58 (19-87)	0.001*^b^
Gender								0.747^a^
Male	30 (20.7)	29 (20.0)	15 (10.3)	5 (3.4)	8 (5.5)	18 (12.4)	40 (27.6)	
Female	20 (17.5)	27 (23.7)	8 (7.0)	4 (3.5)	3 (2.6)	14 (12.3)	38 (33.3)	
Height (cm), range	165 (149-180)	165 (147-184)	167 (151-182)	158 (145-170)	165.5 (156-177)	164.5 (155-180)	166 (150-186)	0.482^b^
Weight (Kg), range	60 (44-87)	61 (40-86)	65 (46-84)	55 (46-70)	58.75 (50-84)	61 (47-83)	61 (42-99)	0.530^b^
BMI (Kg/m^2^), range	22.0 (17.0-22.4)	23.5 (14.7-29.4)	23.7 (16.5-29.0)	22.9 (17.1-25.1)	22.0 (18.7-26.8)	22.0 (18.4-29.3)	22.4 (16.2-33.9)	0.860^b^
B symptoms, No. (%)	14 (20.0)	16 (22.9)	8 (11.4)	4 (5.7)	3 (4.3)	11 (15.7)	14 (20.0)	0.390^a^
Hans typing, No. (%)								0.001*^a^
Non-GCB	38 (22.2)	43 (25.1)	5 (2.9)	6 (3.5)	6 (3.5)	24 (14.0)	49 (28.7)	
GCB	11 (13.4)	12 (14.6)	18 (22.0)	2 (2.4)	5 (6.1)	1 (16.7)	2 (33.3)	
Unclassified	1 (16.7)	1 (16.7)	0 (0.0)	1 (16.7)	0 (0.0)	1 (16.7)	2 (33.3)	
BM involvement, No. (%)	14 (28.0)	9 (18.0)	3 (6.0)	3 (6.0)	5 (10.0)	5 (10.0)	11 (22.0)	0.119^a^
Ann Arbor stage, No. (%)								0.277^a^
I-II	10 (17.5)	14 (24.6)	4 (7.0)	0 (0.0)	0 (0.0)	9 (15.8)	20 (35.1)	
III-IV	40 (20.0)	41 (20.5)	18 (9.0)	9 (3.5)	11 (4.3)	32 (12.5)	58 (29.0)	
IPI score, No. (%)								0.175^a^
0-1	8 (14.5)	11 (20.0)	5 (9.1)	0 (0.0)	2 (3.6)	7 (12.7)	22 (40.0)	
2-3	18 (14.9)	30 (24.8)	11 (9.1)	3 (2.5)	6 (5.0)	16 (13.2)	37 (30.6)	
4-5	20 (27.4)	14 (19.2)	7 (9.6)	3 (2.5)	6 (8.2)	16 (13.2)	15 (20.5)	
WBC (10×10^9^/L), range	5.2 (0.9-11.9)	6.7 (2.2-17.4)	7.0 (1.5-17.3)	3.7 (2.2-14.6)	7.1 (4.2-12.0)	6.6 (2.7-19.2)	5.6 (2.1-30.9)	0.093^b^
Hb (g/L), range	123.5 (46-160)	129 (69-162)	123 (93-174)	123 (57-148)	123 (51-153)	110 (57-156)	128 (54-166)	0.265^b^
PLT (10×10^9^/L), range	171 (29-367)	202.5 (40-404)	229 (59-428)	155 (52-264)	218 (93-541)	227 (20-486)	225 (41-434)	0.163^b^
Ab (g/L), range	39.5 (23-51)	42.1 (24-50)	40.8 (32-48)	41.1 (30-47)	41.3 (29-47)	40.1 (27-53)	41.9 (23-52)	0.292^b^
ALT (U/L), range	17.5 (6-53)	17.5 (6-56)	12 (7-59)	23 (11-65)	15 (7-37)	17 (4-157)	17 (5-60)	0.616^b^
Scr (μmol/L), range	69 (34-168)	75 (32-152)	75 (49-285)	64 (36-133)	77 (55-110)	74 (37-266)	69 (42-193)	0.296^b^
LDH (IU/L), range	291 (94-2752)	329 (139-2259)	352 (131-772)	556 (215-1429)	248 (149-630)	280 (145-10540)	224 (118-2220)	0.034*^b^
Ferritin (ng/mL), range	462.6 (35-5102)	429.4 (24-40000)	355.8 (34-1224)	699.9 (80-12555)	333.9 (12-4533)	288.8 (5-3177)	182.6 (4-1616)	<0.001*^b^
CRP (mg/L), range	10.0 (0-167)	12.7 (0-160)	19.4 (0-120)	27.3 (2-167)	41.3 (2-230)	19.5 (1-118)	5.9 (0-80)	0.082^b^
β2-MG	2665 (0-7460)	2795 (1160-10980)	2470 (1150-16790)	4190 (1990-6500)	3550 (1450-12330)	2470 (1329-14400)	2070 (230-7700)	0.008* ^b^
IL-6 (kU/L), range	14.1 (1.5-716.6)	9.0 (1.5-416.3)	10.5 (0.7-174.6)	10.3 (0.1-136.9)	26.4 (4.0-1436.1)	10.1 (2.9-378.6)	6.0 (0.4-424.3)	0.027* ^b^
IL-10 (kU/L), range	10.0 (0.7-1046.7)	3.9 (0.7-2226.2)	4.6 (0.1-54.8)	39.9 (1.8-6007.0)	5.9(0.1-49.2)	4.6(0.1-512.6)	3.5 (0.1-475.9)	<0.001* ^b^
TG (mmol/L), range	1.3 (0.6-6.2)	1.5 (0.5-3.7)	2.0 (0.5-5.3)	1.2 (0.5-2.6)	1.3 (0.7-2.7)	1.5 (0.6-22.0)	1.1 (0.4-5.4)	0.003* ^b^
TC (mmol/L), range	3.6 (2.2-7.4)	4.1 (1.6-5.7)	4.3 (2.3-6.9)	4.9 (2.5-5.6)	4.2 (2.5-6.2)	4.1 (2.3-6.3)	4.1 (2.1-8.1)	0.177 ^b^
HDL (mmol/L), range	0.9 (0.2-2.7)	1.1 (0.2-1.6)	0.9 (0.6-1.8)	1.1 (0.3-2.2)	0.9 (0.6-1.4)	0.9 (0.2-2.0)	1.0 (0.2-2.0)	0.092 ^b^
LDL (mmol/L), range	2.1 (1.0-4.6)	2.2 (0.1-3.7)	2.3 (0.6-4.3)	2.7 (1.7-3.9)	2.2 (1.0-4.4)	2.3 (0.9-3.5)	2.4 (0.3-5.7)	0.285 ^b^

BMI, Body Mass Index; Non-GCB, non-Germinal Center B-cell; GCB, Germinal Center B-cell; BM, bone marrow; IPI, International Prognostic Index; WBC, white blood cell; Hb, hemoglobin; PLT, platelet; Ab, albumin; ALT, alanine aminotransferase; Scr, serum creatinine; LDH, lactate dehydrogenase; CRP, C-reaction protein; β2-MG, β2-microglobulin; IL-6, Interleukin-6; IL-10, Interleukin-10; TG, triglyceride; TC, total cholesterol; HDL, high-density lipoprotein; LDL, low-density lipoprotein.

*Significantly different.

Continuous variables are presented as median with range and categorical variables are shown as frequency and percentage (No, %).

a Fisher‘s exact test. ^b^Kruskal-Wallis test.

### Other clinical factors about dyslipidemia

Binary logistic regression is used to analyze other clinical elements related to hypertriglyceridemia and dyslipidemia. In univariable analysis, molecular typing, *BCL2* fusion translation, ferritin, and BMI were risk factors for hypertriglyceridemia (p <0.05). In terms of dyslipidemia, B symptoms, molecular typing, *BCL2* fusion translation, ferritin, CRP, and IL-6 have influenced dyslipidemia (p <0.05).

In multivariable analysis, results indicate that being one of the EZB subtypes (p = 0.002, OR 34.524, 95% CI 3.700–322.120) is correlative to hypertriglyceridemia. Besides, the level of BMI affects triglycerides (p = 0.002, OR 1.183, 95% CI 1.064–1.315). As for dyslipidemia, the EZB subtype is significantly associated with dyslipidemia (p = 0.020, OR 14.931, 95% CI 1.532–145.477). The influence of ferritin level is familiar (p = 0.026, OR 1.001, 95% CI 1.000–1.001). The detailed results of the logistic regression model are shown in **Table 2**.

**Table 2:** Logistic regression models evaluating the associations between clinical variables and hypertriglyceridemia and dyslipidemia in DLBCL patients.

Dependent variable	Independent variable	Univariable analysis		Multivariable analysis	
		OR (95% CI)	P-value	OR (95% CI)	P-value
	Age	1.377 (0.816–2.321)	0.231	1.384 (0.715–2.677)	0.335
	Sex	0.989 (0.971–1.006)	0.199	0.879 (0.955–1.001)	0.062
	B symptoms	1.738 (0.989–3.053)	0.054	1.291 (0.627–2.661)	0.488
**HTG**	MCD subtype	3.150 (1.454–6.822)	0.004*	3.751 (0.721–19.507)	0.116
	BN2 subtype	2.362 (1.055–5.292)	0.037*	2.386 (0.907–6.272)	0.078
	EZB subtype	15.120 (4.387–47.260)	<0.001*	34.524 (3.700–322.120)	0.002*
	A53 subtype	2.520 (1.014–6.265)	0.047*	2.741 (0.977–7.692)	0.055
	BCL2 fusion mutation	4.104 (1.309-12.866)	0.015*	0.403 (0.034–4.814)	0.473
	ferritin	1.000 (1.000–1.001)	0.009*	1.000 (1.000–1.001)	0.167
	IL-10	1.001 (1.000–1.002)	0.096	1.001 (1.000–1.003)	0.135
	BMI	1.156 (1.058–1.265)	0.001*	1.183 (1.064–1.315)	0.002*
	Age	1.442 (0.877–2.371)	0.149	1.485 (0.805–2.739)	0.206
	Sex	0.990 (0.973–1.007)	0.238	0.976 (0.955–0.998)	0.035*
**Dyslipidemia**	B symptoms	2.178 (1.226–3.871)	0.008*	1.579 (0.764–3.264)	0.218
	MCD subtype	1.880 (0.938–3.768)	0.075	2.041 (0.405–10.287)	0.387
	BN2 subtype	1.642 (0.802–3.363)	0.175	1.261 (0.524–3.031)	0.605
	EZB subtype	10.108 (2.767–36.919)	<0.001*	14.931 (1.532–145.477)	0.020*
	A53 subtype	2.216 (0.958–5.126)	0.063	1.937 (0.732–5.123)	0.183
	BCL2 fusion mutation	7.232 (1.603–32.628)	0.010*	0.559 (0.041–7.714)	0.664
	ferritin	1.001 (1.000–1.002)	0.001*	1.001 (1.000–1.001)	0.026*
	IL-6	1.006 (1.000–1.009)	0.048*	1.002 (0.998–1.005)	0.306
	CRP	1.013 (1.011–1.019)	0.013*	1.000 (0.991–1.010)	0.979
	BMI	1.079 (0.992–1.172)	0.075	1.080 (0.975–1.195)	0.140

OR, odd ratio; CI, confidence interval; HTG, hypertriglyceridemia; IL-10, interleukin-10; BMI, Body Mass Index; IL-6, Interleukin-6; CRP, C-reactive protein.

*Significantly different.

### Relation between dyslipidemia and survival

**Figure 4:**
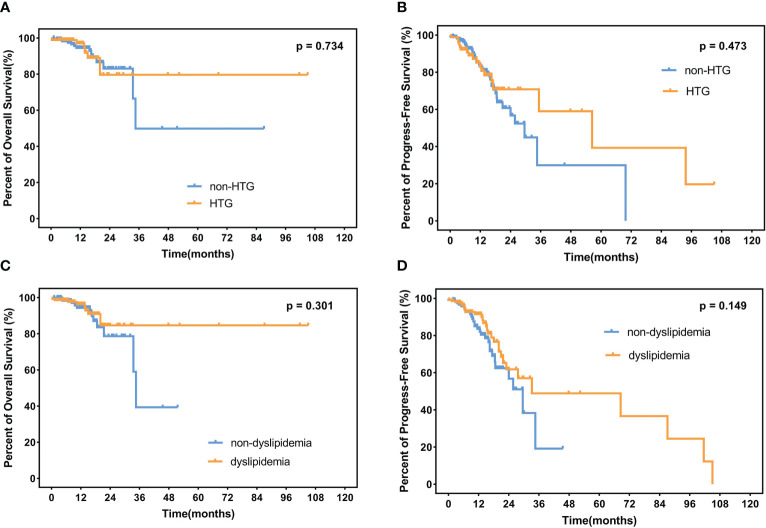
OS and PFS in patients. **(A)** Percent of OS and **(B)** progress-free survival in patients with or without HTG. **(C)** Percent of overall survival and **(D)** progress-free survival in dyslipidemia patients and non-dyslipidemia patients. OS, overall survival; PFS, progress-free survival; HTG, hypertriglyceridemia.

**Figure 5:**
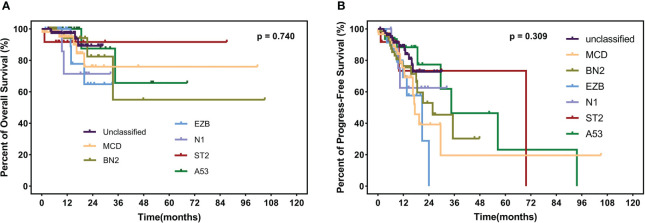
OS **(A)** and PFS **(B)** in different subtypes, including MCD, BN2, EZB, N1, ST2, A53, and unclassified subtypes. OS, overall survival; PFS, progress-free survival.

**Figure 6:**
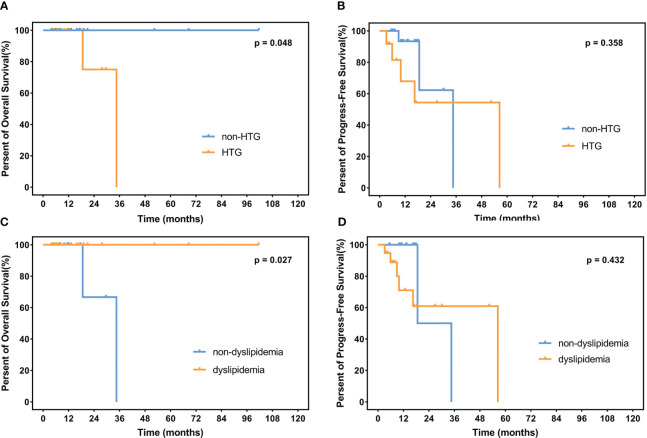
OS and PFS in the A53 subgroup. **(A)** illustrates OS in the HTG group and the non-HTG group with significant statistical differences. **(B)** shows PFS in these two groups without a significant statistical difference. **(C, D)** indicate OS and PFS in the dyslipidemia group and the non-dyslipidemia group, with a significant statistical difference only in OS. OS, overall survival; PFS, progress-free survival. HTG, hypertriglyceridemia.

The median follow-up time is 12.3 months (range 1.1–105.0 months) among the 235 surviving patients. A total of 21 patients died, three of whom died of complications, and 18 patients died of disease progression. Significantly, only two patients died during induction treatment. Moreover, up to the cut-off time, disease progress occurred in 57 patients, and in 35 patients, disease progress occurred at the first assessment during therapy. Three patients were lost to follow-up. The 2-year OS and 2-year PFS rates were 81.7% and 61.3%, respectively. We interpret the influence of lipids on survival. Neither hypertriglyceridemia nor dyslipidemia significantly affected OS and PFS in DLBCL patients (hypertriglyceridemia: OS hazard ratio HR 0.855, 95% CI 0.353–2.075, p = 0.7336; PFS HR 0.824, 95% CI 0.482–1.407, p = 0.4730. dyslipidemia: OS hazard ratio HR 0.637, 95% CI 0.270–1.504, p = 0.3012; PFS HR 0.692, 95% CI 0.408-1.176, p = 0.1490, **Figure 4**). OS and PFS in different genetic subtypes showed no significant statistical divergence (**Figure 5**). Interestingly, OS in the A53 subgroup showed differences. The 32 patients who are defined as the A53 subgroup were divided into the HTG group and the non-HTG group by the level of triglycerides, and they also divided into the dyslipidemia group and the non-dyslipidemia group. Although the small number of samples precludes achieving a significant difference in PFS, the OS showed significant results. The non-HTG group revealed better survival than the HTG group, while the non-dyslipidemia group showed worse survival than the dyslipidemia group (**Figure 6**). Relative parameters that significantly influenced survival were not discovered in either univariable or multivariable Cox regression models (**Table S3**).

## Discussion

In our study, hypertriglyceridemia and dyslipidemia are associated with the molecular typing of DLBCL, and the most relevant is the EZB subtype. The other clinical factors also connect with dyslipidemia, including B symptoms, *BCL2* fusion translation, ferritin, and BMI. We demonstrated the correlation of dyslipidemia with the genetic type of DLBCL at the genetic level. While the incidence of hypertriglyceridemia and dyslipidemia has not influenced OS and PFS. Research shows that prostate cancer patients with high levels of triglycerides have a higher risk of relapse. But in some malignancies, dyslipidemia is not associated with a poor prognosis, such as gastric cancer and breast cancer (18–20). We conjectured that patients who have dyslipidemia were provided therapy to lower lipids, and dyslipidemia is not an independent prognosis factor. Besides, Schmitz et al. reported different OS in MCD, BN2, EZB, and N1 subtypes (3). In our study, OS and PFS did not show statistical differences. We inferred that it might be related to the small size of the patients and not enough follow-up time.

Dyslipidemia is characterized by abnormal serum triglycerides, cholesterol, low-density lipoprotein, and high-density lipoprotein. The Frederickson model was raised to systematically categorize dyslipidemia into five phenotypes. Type 2 is characterized by elevated levels of LDL, and the rest of the types are characterized by increased levels of various triglyceride-rich lipoprotein subfractions (16). A more practical model of dyslipidemia defines it as hypertriglyceridemia, hypercholesterolemia, hypo-HDL, and hyper-LDL. The recommended levels of TG, LDL, and HDL are 2–9.9 mmol/L, 3.4–4.9 mmol/L, and 0.7–0.9 mmol/L (15). Exorbitant levels of lipids express an increased risk of pancreatitis (21). In our retrospective study, two patients had high levels of triglycerides (28.02 and 22.00 mmol/L, respectively). While there were no episodes of acute pancreatitis. Elevated plasma LDL-cholesterol levels are the 8th leading risk factor for death in 2019 worldwide (22). A large study conducted in China indicated that the most common dyslipidemia subtypes are hypo-HDL (20.4%) and HTG (13.8%) (23). The mechanism related to primary dyslipidemia has not been proved definitively. According to genome-wide association studies (GWASes), *TMEM57*, *DOCK7*, *CELSR2*, *APOB*, *ABCG5*, *HMGCR*, *TRIB1*, *FADS2/S3*, *LDLR*, *NCAN*, and *TOMM40-APOE* appear to be related to increased TC levels. Similarly, *CELSR2-PSRC1-SORT1*, *PCSK9*, *NCAN-CILP2-PBX4*, *LDLR*, and *APOC1-APOE* are associated with variations in LDL levels (24). *D9N* and *N291S* variants have been associated with elevated TG, while the *S447X* variant appears to be related to depressed TG. Moreover, *APOA5*-related abnormalities appear to be potentially correlative to TG (25, 26). Besides, pre-B-cell leukemia transcription factor 4 (*PBX4/CILP2* locus) and B-cell CLL/lymphoma 3 genes are considered to be the association factors for dyslipidemia (27, 28). Secondary hyperlipidemia has been acknowledged as being associated with obesity, smoking, the metabolic syndrome, proinflammatory and prothrombotic biomarkers, and type 2 diabetes (29). Recently, the interaction between dyslipidemia-related genes and hematologic malignancy genes has been reported; for instance, the *PML/RARα* fusion protein and peroxisome proliferator-activated receptor-α have a synergistic effect on acute promyelocytic leukemia (30). Besides, the incidence of cardiovascular disease was higher in patients with DLBCL than in normal people (31).

Dyslipidemia has been reported to be associated with a multitude of solid tumors, such as breast cancer, prostate cancer, thyroid cancer, colorectal cancer, and lung cancer (5–9). In terms of hematologic malignancies, chronic lymphocyte leukemia and acute promyelocytic leukemia are reported to be associated with dyslipidemia and hypertriglyceridemia (11, 32). Whereas, the correlation between dyslipidemia and lymphoma or multiple myeloma has not been reported. More recently, studies aimed at lipids in DLBCL patients appeared to indicate a prognosis (33–35). Fundamental experiments investigated cholesterol and DLBCL. *SYK* inhibits cholesterol biosynthesis by modulating *PI3K/AKT*-dependent survival pathways (36). *SOX9*–*DHCR24*–cholesterol biosynthesis axis in *IGH-BCL2* fusion translation positive DLBCL plays an oncogenic role *via* upregulating cholesterol synthesis (12, 37). Based on molecular typing, the EZB subtype is characterized by *IGH-BCL2* fusion translation and the EZH2 mutation. Jiao et al. proposed the *SOX9*–*DHCR24*–cholesterol biosynthesis axis, which may interpret the association between the EZB subtype and dyslipidemia to some extent. *SOX9* was researched more frequently in other solid tumors like colon cancer, hepatocellular carcinoma, and breast cancer. *SOX9* plays an oncogenic role in GCB and *IGH-BCL2*-positive DLBCL. In other words, *SOX9*-positive DLBCLs are more likely to be categorized as EZB subtypes and are more likely to be associated with *NF-κB* signaling. SOX9 upregulates *DHCR24* expression, which is important in the cholesterol biosynthesis pathway (38–40). Other studies indicate that *BCL2* may influence lipid metabolism. Downregulation of *BCL2*-related transcription factors has been considered to affect carotid atherosclerosis and be associated with oxidized LDL trans-differentiation. Thus, we speculate that *BCL2* fusion influences lipid metabolism (41).

The concept of genetic typing was raised to provide potential nosology for precision medicine strategies in DLBCL. According to the research containing 574 DLBCL biopsy samples identified genes, the BN2 subtype is dominated by *NOTCH* pathway aberrations and the *NF-κB* pathway. Besides, the MCD subtype is characterized by *BCR* and *NF-κB* pathway aberrations. EZB subtype is enriched for *BCL2* translocation, *EZH2* mutation, and *REL* amplification, involving Janus-associated kinase–signal transducers and activators of transcription (*JAK-STAT*) signaling and the *PI3K* pathway. *BCR* has been shown to directly promote cholesterol biosynthesis through intermediate kinases downstream of *BCR* to maintain cell membrane integrity and *BCR* signaling (3, 42–46). Aimed at this, targeting cellular cholesterol provides new precise treatment strategies. Thaxton et al. investigated functional lipoprotein nanoparticles in DLBCL via targeting both cellular cholesterol uptake and *BCR*-associated *de novo* cholesterol synthesis and achieved cellular cholesterol reduction and induced apoptosis in otherwise resistant ABC DLBCL cell lines (47).

We analyzed 259 DLBCL patients in the lipid aspect based on genetic typing and deduced the association between dyslipidemia and the EZB subtype without prognosis discrepancy. This study has some deficiencies. Firstly, it is a retrospective study with limitations in patient selection and analysis. Secondly, follow-up times are not long enough because there have been 121 types of gene sequencing analyses conducted in the clinical institution in recent years. Thirdly, the role of dynamic alterations in blood biomarkers during and after treatment was not taken into consideration during the analysis. Further prospective and multicenter research is needed to investigate dyslipidemia in DLBCL to instruct prognosis evaluation and treatment.

## Conclusion

A total of 259 newly diagnosed DLBCL patients were categorized by genetic typing and analyzed for dyslipidemia. Significant differences were found in the EZB subtype; dyslipidemia and hypertriglyceridemia occurred in this isoform without influence on survival. In summary, the EZB subtype is exposed to higher risks of dyslipidemia and hypertriglyceridemia, which may guide clinical treatment strategies.

## Data availability statement

The original contributions presented in the study are included in the article/**Supplementary Material**. Further inquiries can be directed to the corresponding author.

## Ethics statement

The studies involving human participants were reviewed and approved by the Research Ethics Committee of the first affiliated Hospital, College of Medicine, Zhejiang University. Written informed consent was not required to participate in this study in accordance with the national legislation and the institutional requirements

## Author contributions

WX contributed to the conception and design of the article. YX, HS, YS, and YZ wrote the manuscript. XZ, JS, XL, DZ, CY, JHW, XH, JYW, JH, HM, WY, HT, and JJ revised the manuscript. All authors contributed to the article and approved the submitted version.
