# Supplementary material for: Dyslipidemia in diffuse large B-cell lymphoma based on the genetic subtypes: a single-center study of 259 Chinese patients
Source: Front Oncol. 2023 Jun 9;13:1172623. doi: 10.3389/fonc.2023.1172623 (PMC10299728; doi:10.3389/fonc.2023.1172623)
Supplement: Supplementary file 3 [file Table_3.docx]

**Table S3**: Univariable and multivariable Cox proportional hazard regression models for overall survival (OS) in patients with DLBCL.

|  |  |  |  | **Univariable analysis** | | |  | **Multivariable analysis** | | |
| --- | --- | --- | --- | --- | --- | --- | --- | --- | --- | --- |
| **Dependent  variable** |  | **Independent  variable** |  | **OR (95% CI)** |  | **P value** |  | **OR (95% CI)** |  | **P value** |
|  |  | WBC |  | 1.39(0.882-2.192) |  | 0.156 |  | - |  | 0.979 |
| **HTG** |  | PLT |  | 1.014(0.993-1.036) |  | 0.187 |  | - |  | 0.912 |
|  |  | BMI |  | 0.197(0.882-2.192) |  | 0.311 |  | - |  | 0.994 |
|  |  | TG |  | 0.094(0.004-2.292) |  | 0.147 |  | - |  | 0.981 |
|  |  | VLDL |  | 0.006(0.001-11.501) |  | 0.185 |  | - |  | 0.987 |
|  |  |  |  |  |  |  |  |  |  |  |

OR: odd ratio; CI: confidence interval; HTG: hypertriglyceridemia; WBC: white blood cell; PLT: platelet; BMI: Body Mass Index; TG: triglyceride; VLDL: very low-density lipoprotein.

IL-10: Interleukin-10; BMI: Body Mass Index; IL-6: Interleukin-6; CRP: C-reactive protein.

* Significantly different.
